# Virome analysis of two sympatric bat species (*Desmodus rotundus* and *Molossus molossus*) in French Guiana

**DOI:** 10.1371/journal.pone.0186943

**Published:** 2017-11-08

**Authors:** Arielle Salmier, Sourakhata Tirera, Benoit de Thoisy, Alain Franc, Edith Darcissac, Damien Donato, Christiane Bouchier, Vincent Lacoste, Anne Lavergne

**Affiliations:** 1 Laboratoire des Interactions Virus-Hôtes, Institut Pasteur de la Guyane, Cayenne, French Guiana; 2 UMR BIOGECO, Institut National de la Recherche Agronomique (INRA), Cestas, France; 3 Institut Pasteur, Plateforme de Génomique - Pôle Biomics, Paris, France; University of Texas Medical Branch at Galveston, UNITED STATES

## Abstract

Environmental disturbances in the Neotropics (e.g., deforestation, agriculture intensification, urbanization) contribute to an increasing risk of cross-species transmission of microorganisms and to disease outbreaks due to changing ecosystems of reservoir hosts. Although Amazonia encompasses the greatest diversity of reservoir species, the outsized viral population diversity (virome) has yet to be investigated. Here, through a metagenomic approach, we identified 10,991 viral sequences in the saliva and feces of two bat species, *Desmodus rotundus* (hematophagous), trapped in two different caves surrounded by primary lowland forest, and *Molossus molossus* (insectivorous), trapped in forest and urban habitats. These sequences are related to 51 viral families known to infect a wide range of hosts (*i*.*e*., bacteria, plants, insects and vertebrates). Most viruses detected reflected the diet of bat species, with a high proportion of plant and insect-related viral families for *M*. *molossus* and a high proportion of vertebrate-related viral families for *D*. *rotundus*, highlighting its influence in shaping the viral diversity of bats. Lastly, we reconstructed the phylogenetic relationships for five vertebrate-related viral families (*Nairoviridae*, *Circoviridae*, *Retroviridae*, *Herpesviridae*, *Papillomaviridae*). The results showed highly supported clustering with other viral sequences of the same viral family hosted by other bat species, highlighting the potential association of viral diversity with the host’s diet. These findings provide significant insight into viral bat diversity in French Guiana belonging to the Amazonian biome and emphasize that habitats and the host’s dietary ecology may drive the viral diversity in the bat communities investigated.

## Introduction

Bats present a rich viral diversity as compared to other animals and their uniqueness as hosts of pathogenic viruses has animated debates in recent years [1–3]. Their ability to fly over long distances and their diverse feeding strategies seem to facilitate the acquisition and dispersal of viruses across remote regions, as well as cross-species transmissions [2,4,5]. Likewise, their wide variety of both social structures and behaviors contribute to viral transmissions and persistence of viruses in bat populations [2,6]. Studies have also highlighted that bats present long associations with numerous viral families and genera (e.g., *Paramyxoviridae*, *Filoviridae*, *Lyssavirus*, *Henipavirus*), with viruses detected in bats usually being older than those found in humans or other animals [7]. To date, viral metagenomics in bats has almost exclusively focused on North American and Eurasian bat communities and many novel mammalian viruses have been reported, including some that are important to public health [8–17].

Amazonia encompasses a great diversity of bat species. Numerous studies, using conventional methods or high-throughput sequencing, have allowed the identification of a number of viruses in this region [18–21]. For instance, Drexler *et al*. detected and identified a diversity of paramyxoviruses in several New World bat species [22], Tong *et al*. reported novel influenza A viruses in Peruvian flat-faced fruit bats (*Artibeus planirostris*) [23], and Lima *et al*. proposed a new genus for two newly characterized polyomaviruses in *Tadarida brasiliensis* [24].

In French Guiana, an Amazonian region, 106 bat species have been listed [25]. During the last few decades this region has undergone environmental changes (e.g., urbanization, agriculture intensification, deforestation), which have led to alterations in the composition and dynamics of bat communities [26,27]. Studies conducted in the Neotropics reported an overall decrease in species richness and relative abundance correlated with the urban influence, although species highly tolerant to environmental disturbances, such as insectivorous bats, tend to persist in large urban environments [28]. Furthermore, the diversity of bat habitats may influence both microbe transmission and persistence in bat communities. For example, de Thoisy *et al*. [29] showed that pristine forest habitats favored the circulation and maintenance of the rabies virus, compared to disturbed areas, and in Southeast Asian rainforests Gay *et al*. [30] showed that fragmentation reduces both viral and endoparasite species richness.

The aim of our study was to characterize the virome in the feces and the saliva of two abundant and opportunistic Amazonian bat species: the common vampire bat, *Desmodus rotundus* (Phyllostomidae) and Pallas’s mastiff bat, *Molossus molossus* (Molossidae). Both bat species are sympatric in the forest areas of French Guiana. They both have high plasticity and tolerance to perturbations but present distinct diet and social behavior.

*D*. *rotundus* bats are hematophagous and live in groups in highly complex social systems, for which Wilkinson *et al*. showed a strong association between the mating system and genetic structure of populations [31,32]. Studies on *D*. *rotundus* also highlighted that their diet associated with social interactions (especially social grooming and food sharing) within colonies contribute to the transmission and maintenance of viruses such as rabies [29,33]. Furthermore, a modeling study of rabies transmission in a *Desmodus* population in Peru identified metapopulation dynamics as a major driver of rabies maintenance [34]. Turmelle and Olival also pointed out that genetic spatial structure and the host’s ecology and social behavior correlate with viral richness [35]. We investigated the virome of *D*. *rotundus* bats trapped in two caves (F and M) located in forest areas, for which no exchange of individuals was observed through a longitudinal mark-recapture study [29].

*M*. *molossus* bats are insectivorous and live in stable social groups that practice social foraging over large spatial scales [36]. Studies on Molossidae also highlighted that members of this family tend to benefit from increased foraging resources in urban areas and persist in large urban environments for which they present high behavioral flexibility and heterogeneity [28]. We investigated the virome of *M*. *molossus* bats trapped in urban and forest areas.

Lastly, focusing on viral families known to infect mammals, we investigated the phylogenetic relationships of viral sequences related to five viral families (*Circoviridae*, *Herpesviridae*, *Nairoviridae*, *Papillomaviridae* and *Retroviridae* (genus *Spumavirus*)), detected in either high proportion in both species and all habitats or only a distinct bat species and habitat. These findings allowed identifying novel vertebrate-related viral sequences and emphasize the importance of studying the role of habitats and dietary ecology in shaping viral diversity for these two Amazonian bat species.

## Materials and methods

### Ethics statement

All animals were captured, handled and sampled following ASM guidelines [37] under the supervision of researchers granted the French animal experimentation level 1 diploma. Bats are not protected by law in French Guiana. The project was nevertheless submitted to and approved by the Conseil Scientifique Régional pour le Patrimoine Naturel de la Guyane. Captures that occurred within protected areas (nature reserves) received approval by the Conseil Scientifique Régional du Patrimoine Naturel on 26 January 2010 and ad hoc authorizations (No. 2011–35 dated 05/30/2011, No. 35 and 59 obtained 03/21/2013 and 04/17/2013, respectively, and delivered by the Prefecture of French Guiana).

### Study areas

*D*. *rotundus* bats were sampled in two caves (F and M) located 25 km apart in pristine primary lowland forests, presenting differences in physical patterns, cave-inhabiting bat species as well as population size (Table 1 and S1 Fig). Cave F is cohabited by four bat species (*i*.*e*., *Anoura geoffroyi*, *Carollia perspicillata* and *Trachops cirrhosus*), and the *D*. *rotundus* population ranges from 60 to 100 individuals [29]. Cave M is larger and moister than cave F, suggesting different roost characteristics and carrying capacity. Cave M is cohabited by at least nine different bat species (*i*.*e*., *Anoura geoffroyi*, *Artibeus* spp., *Carollia perspicillata*, *Pteronotus rubiginosus* and *P*. *sp3*, *Tonatia saurophila*, *Trachops cirrhosus* and *Xophostoma sylvicola*), and the *D*. *rotundus* population ranges from 120 to 150 individuals [29].

**Table 1:** Characteristics of the sampling sites and number of collected feces and saliva samples for *Molossus molossus* and *Desmodus rotundus*.

Species	Habitats	Sites	GPS coordinates		*n* saliva swabs		*n* feces	
			Latitude (N)	Longitude (W)	Per site	Total	Per site	Total
*D*. *rotundus*	**Forest**	Cave F	4° 38′ 59.144″	52° 17′ 35.441″	0	**0**	75	**75**
		Cave M	4° 32′ 6.82″	52° 9′ 6.239″	50	**50**	66	**66**
					**Total**	**50**	**Total**	**141**
*M*. *molossus*	**Forest**	Paracou	5° 14′ 21.282″	52° 55′ 24.218″	17	**58**	4	**14**
		Saut Athanase	4° 11′ 14.706″	52° 19′ 11.21″	41		10	
	**Urban**	Cacao	4° 34′ 33.771″	52° 28′ 4.926″	18	**30**	0	**5**
		La Chaumière	4° 53′ 5.419″	52° 21′ 4.124″	12		5	
					**Total**	**88**	**Total**	**19**

*M*. *molossus* bats were sampled in two sites located in primary lowland rainforests and under the roofs of houses in two sites located in disturbed areas (Table 1 and S1 Fig). *M*. *molossus* individuals trapped in forest habitats formed monospecific colonies, whereas individuals trapped in urban habitats shared artificial roosts with a congener, *Molossus coibensis*.

### Bat sampling

Feces and saliva samples were collected in both dry and rainy seasons during a 2-year period (April 2012 to April 2014). All animals were trapped with mist nets erected inside roosts or in putative foraging courses. Species were identified on site, using external morphology. Prior to release, 138 saliva swabs were collected from *M*. *molossus* and *D*. *rotundus*, in cave M only (Table 1). Samples were preserved on ice with 500 μl of Dulbecco’s modified Eagle Medium (DMEM, Sigma).

Feces were collected in two ways: *M*. *molossus* bats were held in individual sacks secured to a rope line for at least 10 min, allowing enough time for the excretion of 19 fresh feces of about 100 mg each (*n* = 5 in urban habitats, *n* = 14 in forest habitats; Table 1), while clean plastic sheets were laid down on flat surfaces beneath known roosts for *D*. *rotundus*. Then 141 freshly produced feces of about 150 mg each were collected (*n* = 75 in cave F, *n* = 66 in cave M; Table 1) the following morning and temporarily preserved on ice. All samples were later stored at −80°C.

### Sample processing

Feces and saliva samples were pooled according to the habitats and the species regardless of the season and year, corresponding to four pools for *M*. *molossus* (urban saliva, urban feces, forest saliva, forest feces) and three pools for *D*. *rotundus* (cave F feces, cave M saliva, cave M feces) (Table 1).

Samples were processed as previously described by Victoria *et al*. [38]. Briefly, feces were vigorously homogenized with 5–10 mL DMEM. Samples were cleared of debris by low-speed centrifugation (5 min, 10,000 *g*, 4°C). Eukaryotic and prokaryotic cell-sized particles were removed from supernatants through three successive filtrations (0.8 μm, 0.45 μm and 0.22 μm), using cellulose acetate membrane filters (Nalgene). A differential centrifugation procedure, described by Prescott *et al*. [39], was used to pellet the viral particles. Briefly, filtrates were cleared of smaller and less dense components through a 1-h ultracentrifugation (100,000 *g*, 4°C) procedure. Then the pellets were resuspended in nuclease-free water and cleared of persistent high-density particles with a low-speed centrifugation (15 min, 10,000 *g*, 4°C). Lastly, viral particles were pelleted with a 1-h ultracentrifugation step (100,000 *g*, 4°C).

For saliva samples, swabs were vigorously resuspended and 200 μL of suspension from each collecting tube was used to constitute the pools. Samples were cleared of debris by low-speed centrifugation (5 min, 10,000 *g*, 4°C). Eukaryotic and prokaryotic cell-sized particles were removed from supernatants through two successive filtrations (0.45 μm and 0.22 μm), using cellulose acetate membrane filters (Nalgene). The filtrates were cleared of persistent high-density particles with low-speed centrifugation (15 min, 10,000 *g*, 4°C), then viral particles were pelleted with a 1-h ultracentrifugation step (100,000 *g*, 4°C). All viral pellets were resuspended in 40 μL of nuclease-free water.

### Nuclease treatment and viral acid nucleic extraction

Resuspended viral pellets from feces were treated with a mixture of DNases (Turbo DNase from Ambion and Benzonase from Novagen) and RNase One (Promega) to digest nonenveloped nucleic acids (*i*.*e*., those not in viral capsids) [40]. Resuspended viral pellets from saliva samples were treated with the mixture of DNases. All viral nucleic acids (RNA and DNA) were then extracted using the NucliSENS easyMAG^®^ bio-robot (bioMérieux).

### Reverse transcription and amplification

For both feces and saliva samples, the RNA virus-only and DNA virus-only libraries were respectively constructed using a whole transcriptome (WTA) or a whole genome (WGA) amplification method previously described by Berthet *et al*. [41].

For the RNA virus-only amplification, an aliquot of the extracted viral nucleic acid collected was treated with Turbo DNase to remove viral DNA. Persistent rRNA was depleted with the GeneRead^™^ rRNA depletion kit (Qiagen), following the manufacturer’s recommendations. Then viral RNA amplification was performed as described in the protocol of the QuantiTect^®^ Whole Transcriptome Kit (Qiagen) except for the reverse transcription step. cDNA was synthesized using SuperScript^®^ III Reverse Transcriptase (Invitrogen) and random hexamers (Roche), following the manufacturers’ recommendations.

For DNA virus-only amplification, an aliquot of the extracted viral nucleic acid collected was treated with RNase One to remove viral RNA. Then viral DNA amplification was performed as described in the protocol of the QuantiTect^®^ Whole Genome Kit (Qiagen). To ensure homogeneity and blunt ends at the end of the strands produced, 10 U of Klenow polymerase (Roche) was added to the WTA- and WGA-amplified nucleic acids with 8 μL of random hexamers (Roche), incubated 1 h at 37°C, followed by 10 min at 75°C. Samples were assayed with a Qubit^®^ fluorometric quantitation (Qiagen), using the dsDNA Broad Range and dsDNA High Sensitivity Qubit^®^ assay kits, as recommended by the manufacturer.

### High-throughput sequencing

For both feces and saliva samples, 1 μg of each viral library was pooled together, whenever possible, to construct RNA plus DNA viral libraries. High-throughput sequencing was carried out at the Genomics platform at the Institut Pasteur, Paris. Shotgun libraries were prepared by standard Illumina protocols using 1 μg of total genomic DNA. Each sample (feces or saliva) was indexed according to its provenance (species and habitats) using Illumina adaptor-specific primers. Samples were sequenced on a MiSeq sequencer in 300-base paired-end reads.

### Bioinformatic pipeline

**Fig 1:**
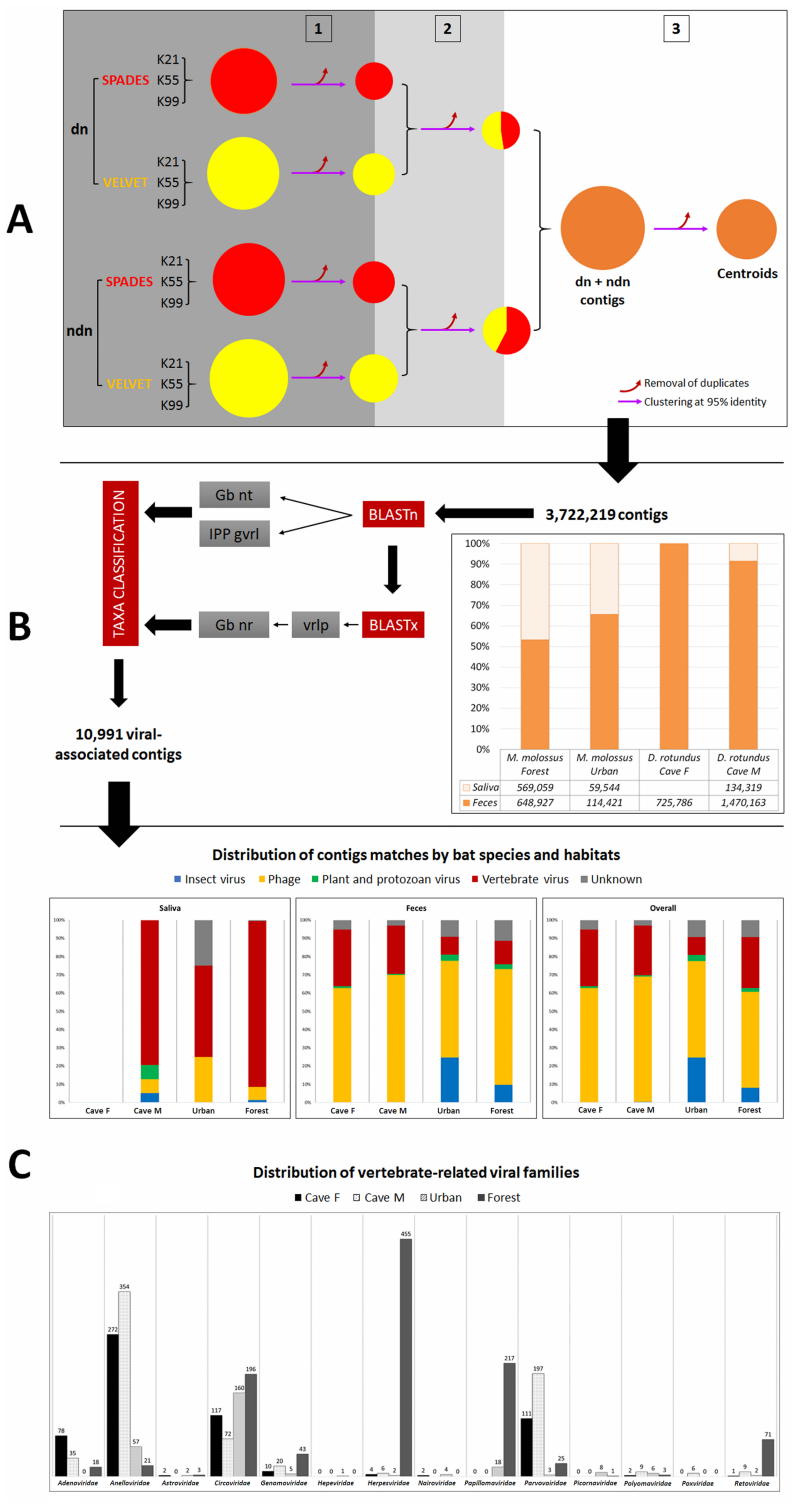
Bioinformatic analysis pipeline for the 3,722,219 assembled contigs, based on Baker *et al*. [45]. (A) Contigs assembled from both normalized and non-normalized sequence reads by two *de novo* assemblers (SPAdes and Velvet) were consolidated by sequential comparisons (numbered shadowed areas) and removal of duplicate sequences (red arrows). (B) Centroids subjected to sequential BLAST comparison and manually controlled taxonomic classification to identify viral sequences. Proportion of sequences assembled from feces (red) and saliva swabs (blue) are shown in the stacked chart for each species depending on habitats (caves F and M for *D*. *rotundus* and Urban and Forest for *M*. *molossus*). (C) Column charts represent the distribution of contig matches (per pooled samples and overall) and the distribution of vertebrate-related viral families by bat species and habitats.

The Illumina sequencing reads were grouped according to their adaptor tags and were processed individually. Sequence files were stripped of their adapter sequences using Trimmomatic v.0.32 [42]. Duplicate sequence reads were removed with FastqMcf v.1.04.676 and reads were quality-filtered using the fastq_quality_filter program from the Fastx toolkit v.0.0.13 (available at http://hannonlab.cshl.edu/fastx_toolkit/index.html), with a quality threshold of 30 and length criteria of 70%. All cleaned data sets were saved for further analysis. Simultaneously, a three-pass digital normalization was run on each sample file using the Khmer software library [43,44]. Normalized files were saved for further analysis. Following Baker’s contig consolidation strategy [45], both normalized and non-normalized reads were *de novo* assembled using two assembly algorithms: Velvet [46] and SPAdes [47]. Three k-mer sizes (21, 55 and 99) were used for both assemblers. The contigs generated were compared (Fig 1) between k-mer sizes for each assembler (comparison 1), between assemblers (comparison 2) and between normalized and non-normalized data sets (comparison 3). A removal of duplicated contigs and a clustering, using Usearch v.7.0.1090 (available at http://www.drive5.com/usearch, [48]) with a 95% threshold identity value, were performed following each comparison. Clustered contigs from comparison 3 (centroids) were considered as consolidated contig sets with lengths ranging from 33 to 45,057 bp and low redundancy. Centroids were submitted to BLASTn comparison with both the NCBI nt and Institut Pasteur de Paris gbvrl databases (in March 2015), (Fig 1). The best-matched reference sequence was retrieved from both databases (cutoff value: 10^e-5^). A length comparison between the results was performed using a bit score ratio (br = bitscore_blastn_nt/bitscore_blastn_gb_vrl), and only contigs with br > 0.90 were regarded as suspect-viral sequences and conserved for further analysis. Centroids not identified as suspect-viral underwent a BLASTx comparison against a custom-made database, which included all protein sequences of the viral kingdom (cutoff value: 10^e-3^). To reduce the misidentification bias of false-positive sequences, identified suspect-viral sequences underwent a second BLASTx comparison with the NCBI nr database (cutoff value: 10^e-3^) (Fig 1). Sequences not retained were discarded. Taxonomic information and the kingdom of each gene id were retrieved with taxdb.

### Phylogenetic analysis

Contigs from five vertebrate-related viral families: *Nairoviridae*, *Circoviridae*, *Retroviridae*, *Herpesviridae* and *Papillomaviridae* were used for phylogenetic analysis. Reference genomes or nucleotide sequences of previously identified viruses were downloaded from GenBank. The accession number of viral sequences used to infer the phylogenetic trees are given in the respective analyses. Nucleotide and protein sequence editing were performed with Geneious R9 (available at http://www.geneious.com, [49]). Sequences were aligned using the Mafft alignment tool [50] included in the software. Nucleotide and protein sequences were trimmed and gap-stripped prior to phylogenetic analyses. For each analysis, the best-fitted model of nucleotide or amino acid substitution was selected using jModelTest 2 [51] and ProtTest 3 [52], respectively, under corrected Akaike information criteria (AICc). Bayesian phylogenetic analyses were performed using MrBayes 3. The Markov chain Monte Carlo (MCMC) algorithm was run with four chains with 10 million generations each, with trees sampled every 500 generations and a burn-in of 25%. Validation of the inference was assessed based on the standard deviation of split frequencies, less than the expected threshold value of 0.01 in MrBayes and by inspecting the effective sampling size (ESS > 500) criterion in Tracer version 1.6 [53].

### Nucleotide sequence accession numbers

All virus sequences reported in this study were deposited in the GenBank nucleotide database under accession numbers KX812440 to KX812444, KX812446, KX812447, KX821677 and KX954092. The data from Illumina sequencing were deposited in GenBank Sequence Reads Archive under accession numbers SAMN05725475−SAMN05725481.

## Results

### Illumina sequencing and assembly

**Fig 2:**
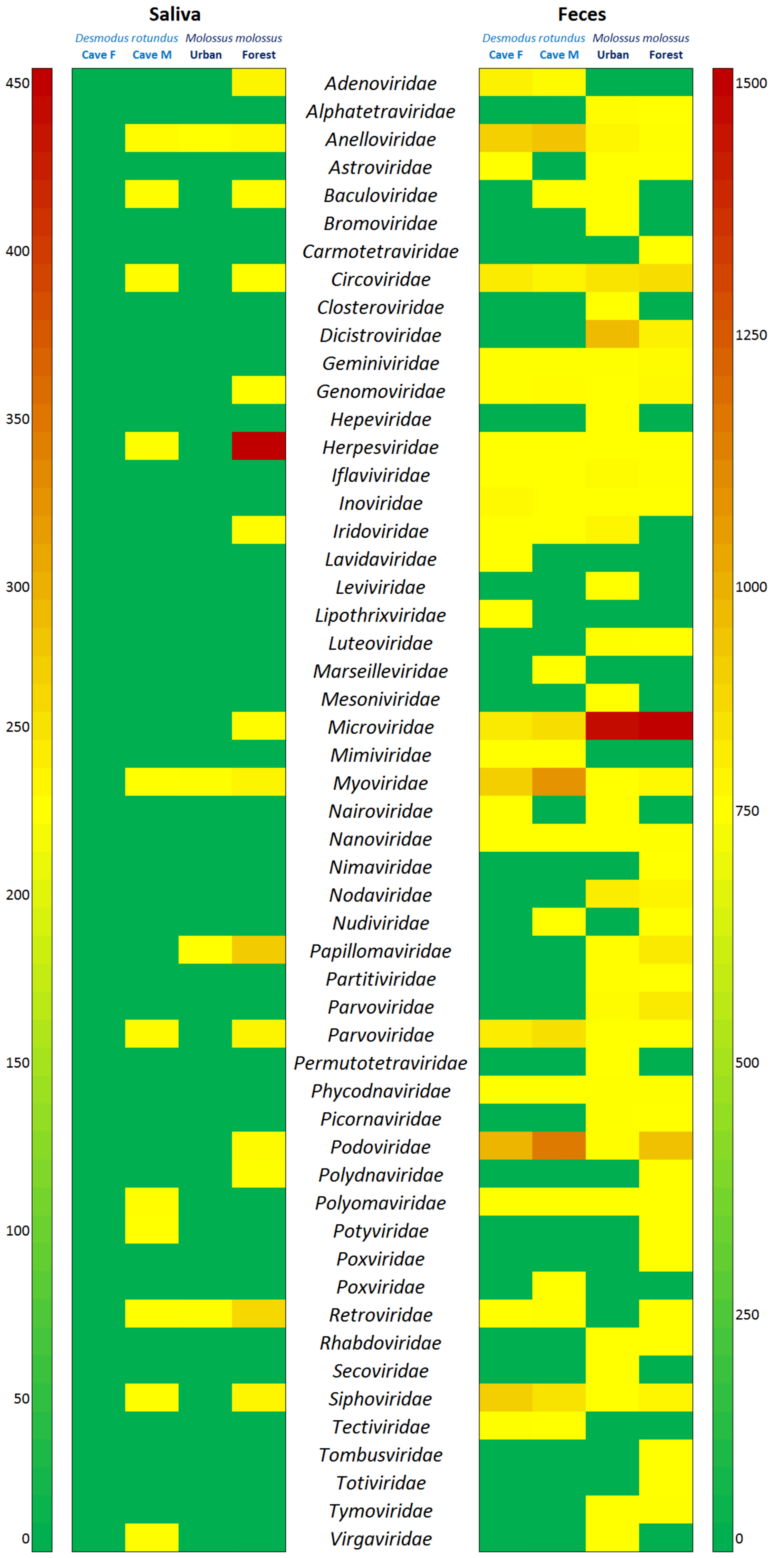
Heatmap based on the viral-associated contigs of 51 families of insect, phage, plant/protozoan and vertebrate viruses in each pooled sample. Location information is provided above each column (Caves F and M for *D*. *rotundus*, Urban and Forest for *M*. *molossus*). The names of the viral families are presented in alphabetical order in the middle. The boxes colored from green to dark red represent the number of contigs observed. Contigs varied between 1 and 450 for the saliva samples and between 1 and 1465 for feces. The scales are given for each type of sample.

Overall, we obtained 53,325,594 raw read sequences (Table A in S1 File). For *M*. *molossus*, 33,333,557 raw sequences were generated from both feces and saliva samples and 19,992,037 for *D*. *rotundus*. Following the data reduction steps, 10,339,752 non-digitally normalized reads and 4,117,544 digitally normalized reads were used for *de novo* assembly. SPAdes and Velvet assemblers generated a comparable number of contigs, even between normalized and non-normalized data. Between 50.22% and 67.66% of the contigs were saved after comparison 1 (Table B in S1 File). Following comparison 2, between 68.09% and 73.19% of the contigs from comparison 1 were saved for further analyses (Table C in S1 File). Finally, between 50.61% and 56.30% of the contigs from comparison 2 were saved to generate the centroids (Table D in S1 File). Following our consolidation steps, a total of 3,722,219 contigs were used for the taxonomic assignment. Most of the contigs retained originated from feces (Fig 1). *Eukaryota*-, *Bacteria*- and *Virus*-associated contigs accounted for 1.02%, 3.82% and 0.30% of the total of consolidated contigs, respectively (Table E in S1 File). A total of 10,991 viral-associated sequences related to 51 known viral families were identified based on their most significant BLAST matches (Fig 2). The total number of virus-associated contigs ranged from 1,930 for *D*. *rotundus* in cave F to 3,768 for *M*. *molossus* in forest areas (Table F in S1 File).

### General virome information and phage-related sequences

More than half of the viral-associated contigs matched phage sequences (Fig 1 and Table 2), accounting for nine phage families belonging to the dsDNA, ssDNA and ssRNA positive-strand groups. These families were identified in both feces and saliva samples (Fig 2 and Table 2). Among the nine phage families, the *Podoviridae* family of the dsDNA order *Caudovirales* accounted for greatest number of phage sequences detected in *D*. *rotundus* while the *Microviridae* family accounted for most of the phage sequences identified for *M*. *molossus* (Fig 2 and Table 2). The eukaryotic viral sequences (insect, plant/protozoan and vertebrate viruses) accounted for about three-tenths of the total of the viral-associated contigs identified, representing 42 known viral families. A high proportion of these families reflected the diet of bats, especially for *M*. *molossus* trapped in urban areas where most of the eukaryote-associated viruses identified belonged to nonmammalian viruses (Fig 1 and Table 2). Contigs matching unclassified viral sequences accounted for one-tenth of the total of viral contigs (Fig 1 and Table 2).

**Table 2:** Contingency table of the viral families identified in the feces and saliva samples of *D*. *rotundus* and *M*. *molossus*.

	**Order**	**Viral family**	**Genome**	***Desmodus rotundus***						***Molossus molossus***						**Overall total**
				**Cave F**			**Cave M**			**Urban**			**Forest**			
				**Saliva**	**Feces**	**Total**	**Saliva**	**Feces**	**Total**	**Saliva**	**Feces**	**Total**	**Saliva**	**Feces**	**Total**	
Insect virus	*Nidovirales*	*Mesoniviridae*	ssRNA(+)		0	**0**	0	0	**0**	0	2	**2**	0	0	**0**	**2**
	*Picronavirales*	*Dicistroviridae*	ssRNA(+)		0	**0**	0	0	**0**	0	394	**394**	0	77	**77**	**471**
	*Picornavirales*	*Iflaviviridae*	ssRNA(+)		2	**2**	0	2	**2**	0	32	**32**	0	10	**10**	**46**
	Unassigned	*Alphatetraviridae*	ssRNA(+)		0	**0**	0	0	**0**	0	20	**20**	0	1	**1**	**21**
	Unassigned	*Baculoviridae*	dsDNA		0	**0**	2	1	**3**	0	1	**1**	4	0	**4**	**8**
	Unassigned	*Carmotetraviridae*	ssRNA(+)		0	**0**	0	0	**0**	0	0	**0**	0	1	**1**	**1**
	Unassigned	*Iridoviridae*	dsDNA		1	**1**	0	3	**3**	0	57	**57**	4	0	**4**	**65**
	Unassigned	*Nimaviridae*	dsDNA		0	**0**	0	0	**0**	0	0	**0**	0	4	**4**	**4**
	Unassigned	*Nodaviridae*	ssRNA(+)		0	**0**	0	0	**0**	0	105	**105**	0	62	**62**	**167**
	Unassigned	*Nudiviridae*	dsDNA		0	**0**	0	1	**1**	0	0	**0**	0	1	**1**	**2**
	Unassigned	*Parvoviridae*	ssDNA(+/-)		0	**0**	0	0	**0**	0	28	**28**	17	122	**139**	**167**
	Unassigned	*Permutotetraviridae*	ssRNA(+)		0	**0**	0	0	**0**	0	2	**2**	0	0	**0**	**2**
	Unassigned	*Polydnaviridae*	dsDNA		0	**0**	0	0	**0**	0	0	**0**	2	4	**6**	**6**
	Unassigned	*Poxviridae*	dsDNA		0	**0**	0	0	**0**	0	0	**0**	0	3	**3**	**3**
	Unassigned	Unclassified viruses	-		0	**0**	0	0	**0**	0	23	**23**	0	11	**11**	**34**
Phage	*Caudovirales*	*Myoviridae*	dsDNA		279	**279**	1	617	**618**	2	1	**3**	19	42	**61**	**961**
	*Caudovirales*	*Podoviridae*	dsDNA		435	**435**	0	767	**767**	0	15	**15**	8	361	**369**	**1586**
	*Caudovirales*	*Siphoviridae*	dsDNA		285	**285**	2	173	**175**	0	5	**5**	16	56	**72**	**537**
	*Ligmaenvirales*	*Lipothrixviridae*	dsDNA		1	**1**	0	0	**0**	0	0	**0**	0	0	**0**	**1**
	Unassigned	*Inoviridae*	ssDNA(+)		39	**39**	0	1	**1**	0	1	**1**	0	3	**3**	**44**
	Unassigned	*Lavidaviridae*	dsDNA		4	**4**	0	0	**0**	0	0	**0**	0	0	**0**	**4**
	Unassigned	*Leviviridae*	ssRNA(+)		0	**0**	0	0	**0**	0	3	**3**	0	0	**0**	**3**
	Unassigned	*Microviridae*	ssDNA(+)		132	**132**	0	196	**196**	0	1 402	**1 402**	7	1 465	**1 472**	**3202**
	Unassigned	*Tectiviridae*	dsDNA		7	**7**	0	3	**3**	0	0	**0**	0	0	**0**	**10**
	Unassigned	Unclassified Caudovirales	dsDNA		14	**14**	0	2	**2**	0	0	**0**	0	0	**0**	**16**
	Unassigned	Unclassified dsDNA phages	dsDNA		13	**13**	0	20	**20**	0	0	**0**	2	0	**2**	**35**
Plant and protozoan virus	*Mononegavirales*	*Rhabdoviridae*	ssRNA(-)		0	**0**	0	0	**0**	0	2	**2**	0	2	**2**	**4**
	*Picornavirales*	*Secoviridae*	ssRNA(+)		0	**0**	0	0	**0**	0	2	**2**	0	0	**0**	**2**
	*Tymovirales*	*Tymoviridae*	ssRNA(+)		0	**0**	0	0	**0**	0	1	**1**	0	7	**7**	**8**
	Unassigned	*Bromoviridae*	ssRNA(+)		0	**0**	0	0	**0**	0	5	**5**	0	0	**0**	**5**
	Unassigned	*Closteroviridae*	ssRNA(+)		0	**0**	0	0	**0**	0	1	**1**	0	0	**0**	**1**
	Unassigned	*Geminiviridae*	ssDNA(+/-)		9	**9**	0	10	**10**	0	7	**7**	0	27	**27**	**53**
	Unassigned	*Luteoviridae*	ssRNA(+)		0	**0**	0	0	**0**	0	9	**9**	0	2	**2**	**11**
	Unassigned	Marseilleviridae	dsDNA		0	**0**	0	1	**1**	0	0	**0**	0	0	**0**	**1**
	**Order**	**Viral family**	**Genome**	***Desmodus rotundus***						***Molossus molossus***						**Overall total**
				**Cave F**			**Cave M**			**Urban**			**Forest**			
				**Saliva**	**Feces**	**Total**	**Saliva**	**Feces**	**Total**	**Saliva**	**Feces**	**Total**	**Saliva**	**Feces**	**Total**	
Plant and protozoan virus	Unassigned	Mimiviridae	dsDNA		1	**1**	0	1	**1**	0	0	**0**	0	0	**0**	**2**
	Unassigned	*Nanoviridae*	ssDNA(+)		3	**3**	0	1	**1**	0	2	**2**	0	7	**7**	**13**
	Unassigned	*Partitiviridae*	dsRNA		0	**0**	0	0	**0**	0	17	**17**	0	4	**4**	**21**
	Unassigned	*Phycodnaviridae*	dsDNA		6	**6**	0	2	**2**	0	7	**7**	0	9	**9**	**24**
	Unassigned	*Potyviridae*	ssRNA(+)		0	**0**	1	0	**1**	0	0	**0**	0	1	**1**	**2**
	Unassigned	*Tombusviridae*	ssRNA(+)		0	**0**	0	0	**0**	0	0	**0**	0	2	**2**	**2**
	Unassigned	*Totiviridae*	dsRNA		0	**0**	0	0	**0**	0	0	**0**	0	2	**2**	**2**
	Unassigned	*Virgaviridae*	ssRNA(+)		0	**0**	2	0	**2**	0	5	**5**	0	0	**0**	**7**
	Unassigned	*Unassigned*	-		0	**0**	0	0	**0**	0	32	**32**	0	17	**17**	**49**
Vertebrate virus	*Bunyavirales*	*Nairoviridae*	ssRNA(-)		2	**2**	0	0	**0**	0	4	**4**	0	0	**0**	**6**
	*Herpesvirales*	*Herpesviridae*	dsDNA		4	**4**	3	3	**6**	0	2	**2**	450	5	**455**	**467**
	*Picornavirales*	*Picornaviridae*	ssRNA(+)		0	**0**	0	0	**0**	0	8	**8**	0	1	**1**	**9**
	Unassigned	*Adenoviridae*	dsDNA		78	**78**	0	35	**35**	0	0	**0**	18	0	**18**	**131**
	Unassigned	*Anelloviridae*	ssDNA(-)		272	**272**	6	348	**354**	1	56	**57**	12	9	**21**	**704**
	Unassigned	*Astroviridae*	ssRNA(+)		2	**2**	0	0	**0**	0	2	**2**	0	3	**3**	**7**
	Unassigned	*Circoviridae*	ssDNA(+/-)		117	**117**	7	65	**72**	0	160	**160**	1	195	**196**	**545**
	Unassigned	*Genomoviridae*	ssDNA(+/-)		10	**10**	0	20	**20**	0	5	**5**	1	42	**43**	**78**
	Unassigned	*Hepeviridae*	ssRNA(+)		0	**0**	0	0	**0**	0	1	**1**	0	0	**0**	**1**
	Unassigned	*Papillomaviridae*	dsDNA		0	**0**	0	0	**0**	1	17	**18**	93	124	**217**	**235**
	Unassigned	*Parvoviridae*	ssDNA(+/-)		111	**111**	10	187	**197**	0	3	**3**	2	6	**25**	**319**
	Unassigned	*Polyomaviridae*	dsDNA		2	**2**	4	5	**9**	0	6	**6**	0	3	**3**	**20**
	Unassigned	*Poxviridae*	dsDNA		0	**0**	0	6	**6**	0	0	**0**	0	0	**0**	**6**
	Unassigned	*Retroviridae*	ssRNA-RT		1	**1**	1	8	**9**	2	0	**2**	69	2	**71**	**83**
ND	Unassigned	Environmental samples	-		55	**55**	0	49	**49**	2	166	**168**	0	235	**235**	**507**
	Unassigned	Unclassified ssDNA viruses	ssDNA		45	**45**	0	15	**15**	0	78	**78**	4	106	**110**	**248**
	Unassigned	Unclassified viruses	-		0	**0**	0	12	**12**	0	3	**3**	0	5	**5**	**20**
			**Total**		**1 930**	**1 930**	**39**	**2 554**	**2 593**	**8**	**2 692**	**2 700**	**729**	**3 039**	**3 768**	**10 991**

The number of viral-associated contig sequences is given for each sample (**saliva** and **feces**), **overall**, as well as for each habitat (**caves F** and **M** for *D*. *rotundus*, **urban** and **forest** for *M*. *molossus*). The total number of contigs detected for each collecting site is given in bold.

### Eukaryotic viral sequences

#### Insect viruses

Fourteen insect-related viral families and a group of unclassified viruses were identified (Table 2). Positive single-stranded RNA (ssRNA (+)) viruses were predominant for *M*. *molossus* trapped in urban habitats, with most of the viral sequences identified related to the *Dicistroviridae*, *Nodaviridae* and *Iflaviridae* families. DNA viruses detected in this sample were related to viruses from the double-stranded DNA (dsDNA) *Iridoviridae* family and the single-stranded DNA (ssDNA) *Parvoviridae* family. In contrast, ssDNA viruses (mostly from *Parvoviridae*) were slightly more numerous for *M*. *molossus* trapped in forest habitats compared to RNA viruses mostly from the *Dicistroviridae* and *Nodaviridae* families. The four viral families (*Baculoviridae*, *Iridoviridae*, *Nudiviridae* and *Iflaviridae*) detected for the *D*. *rotundus* were likely related to insects flying around feces.

#### Plant and protozoan viruses

Sixteen plant- and protozoan-related viral families and a group of unassigned viruses were identified (Table 2). ssRNA viruses, mostly from the unassigned group followed by the *Luteoviridae*, *Bromoviridae* and *Virgaviridae* families, were predominant for *M*. *molossus* trapped in urban habitats. In contrast, dsDNA viruses (*Phycodnaviridae*) and ssDNA viruses (*Geminiviridae*) were predominant for *M*. *molossus* and *D*. *rotundus* trapped in forest habitats.

#### Vertebrate viruses

Fourteen vertebrate-related viral families were identified (Fig 1 and Table 2). The highest viral diversity was found in both feces samples and forest habitats, and DNA viruses were the most frequently found viruses for both species. The *Anelloviridae*, *Circoviridae* and *Parvoviridae* families accounted for the highest proportion of DNA viruses for both samples of *D*. *rotundus*, with variation in number according to the sampling sites.

The *Circoviridae* and *Anelloviridae* families were also the most abundant in the sample of *M*. *molossus* trapped in urban habitats, with very few viral sequences detected in saliva. In contrast, for *M*. *molossus* trapped in forest habitats, viral sequences related to the *Herpesviridae*, *Papillomaviridae* and *Circoviridae* families were the most numerous, with herpesviruses found for the most part in the saliva sample.

We found viral sequences related to *Poxviridae* in both species, but only the *D*. *rotundus* trapped in cave M presented mammal-related poxviruses. These sequences presented high amino acid homology (>90%) with the *bovine papular stomatitis virus*, suggesting that this virus came directly from cattle and its presence was related to the dietary habit of *D*. *rotundus* (*i*.*e*., *D*. *rotundus* feeds off the blood of cattle).

RNA viruses were found in distinct bat species or habitats and belonged to the *Astroviridae*, *Nairoviridae*, *Hepeviridae*, *Picornaviridae* and *Retroviridae* families. Astroviruses were only found in *D*. *rotundus* trapped in cave F but in both habitats for *M*. *molossus*. In contrast, nairoviruses were only found in *D*. *rotundus* trapped in cave F and in *M*. *molossus* trapped in forest habitats. Hepeviruses were only found in *M*. *molossus* trapped in urban areas. Picornaviruses were found in both habitats for *M*. *molossus*, but the only sequence detected in forest habitats was different from those detected in urban habitats.

### Viral characterization and phylogenetic relationships of selected viruses

#### Bat circoviruses

**Fig 3:**
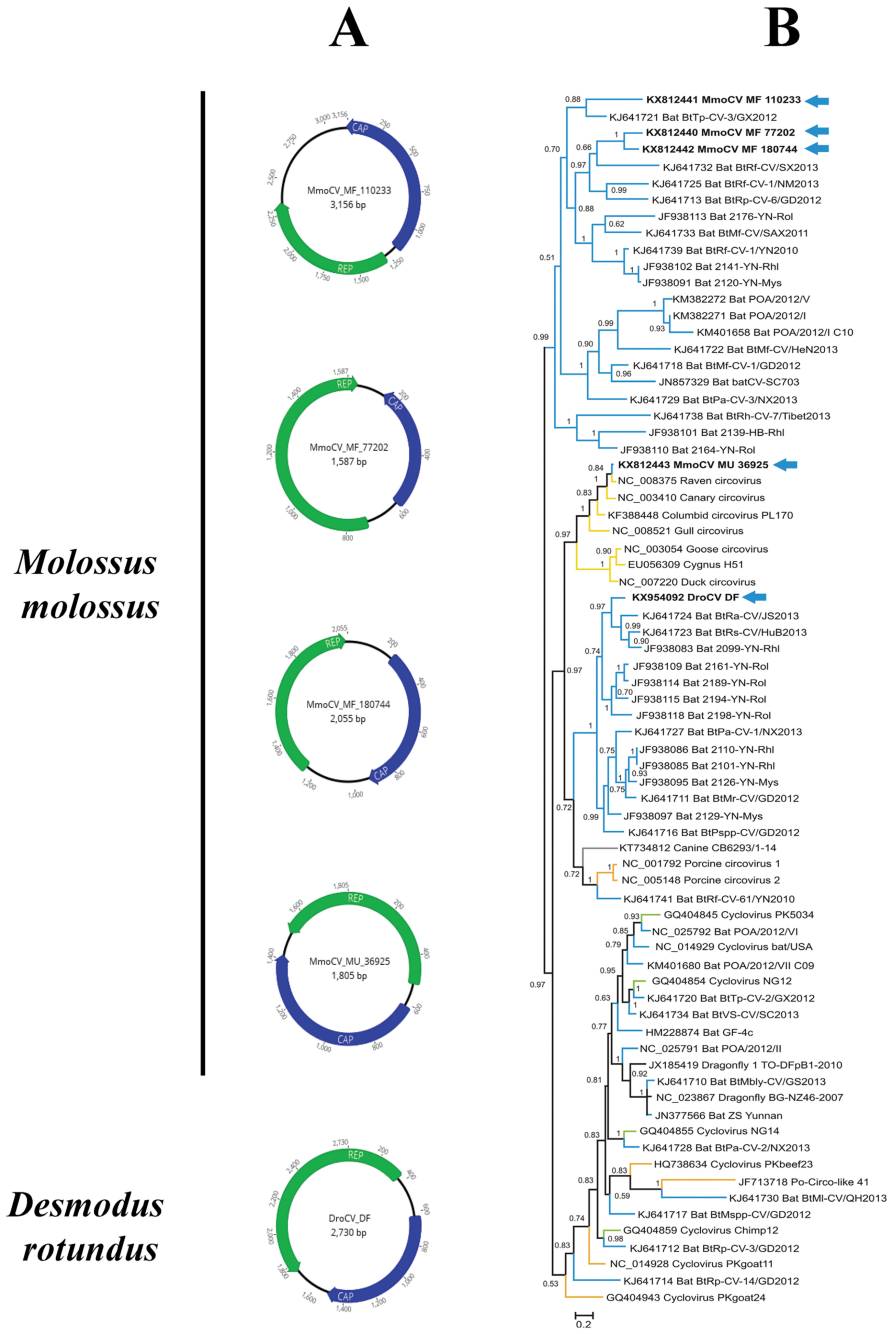
Circular genome maps of five putative circoviruses directly recovered from metagenomic data of *M*. *molossus* and *D*. *rotundus* bat feces and their phylogenetic relationships with other representative members of the *Circoviridae* family. (A) The inversely arranged open-reading frames encoding the putative replication-associated protein (REP) and capsid protein (CAP) are shown in green and blue boxes, respectively. The genome organization was determined with Geneious R9. (B) The phylogenetic analysis is based on the REP protein sequences (alignment of 109 amino acids). The blue arrows indicate the five REP sequences of bat-sourced circoviruses obtained in the present study. The tree was inferred using the Bayesian method with the blosum62 model. Sequence identifiers include the NCBI accession number and the isolate name. Posterior probabilities of the Bayesian analysis (>50%) are shown next to each node. The scale bar indicates amino acid substitutions per site.

The family *Circoviridae* is known to infect birds, mammals and insects, and comprises two genera, *Circovirus* and *Cyclovirus* (ICTV 2016). Their transmission occurs primarily through the fecal–oral route [54]. Here, *Circoviridae* viruses were detected in both *D*. *rotundus* and *M*. *molossus* samples, whatever the environment, with a total of 545 contigs (Table 2). These viruses were essentially found in the feces, except for *D*. *rotundus* in cave M for which a small proportion of sequences was also detected in saliva. Four complete genomes of circoviruses (CVs) were obtained for *M*. *molossus* (*n* = 1 in urban habitats and *n* = 3 in forest habitats), and one partial sequence corresponding to the complete replication-associated protein gene for *D*. *rotundus* in cave M (*n* = 1) (Genbank acc. nb.: KX812440−KX812443 and KX954092). The complete genomes presented the archetypal genome organization of CVs, with the two inversely arranged *ORF*s encoding the Rep and Cap proteins (Fig 3). The intergenic region was variable in length, depending on the species as well as the habitat. The full-length REP was used to perform evolutionary analyses. The phylogenetic tree revealed that CVs (identified in cave M for *D*. *rotundus* and forest habitats for *M*. *molossus*) formed two distinct monophyletic clades with other bat circoviruses detected in the gastrointestinal tract and feces samples of Chinese bat species [55] (Fig 3). In contrast, the CV identified in urban areas for *M*. *molossus* was closely related to CVs detected in birds, with a posterior probability of 0.84.

#### Bat spumaviruses

**Fig 4:**
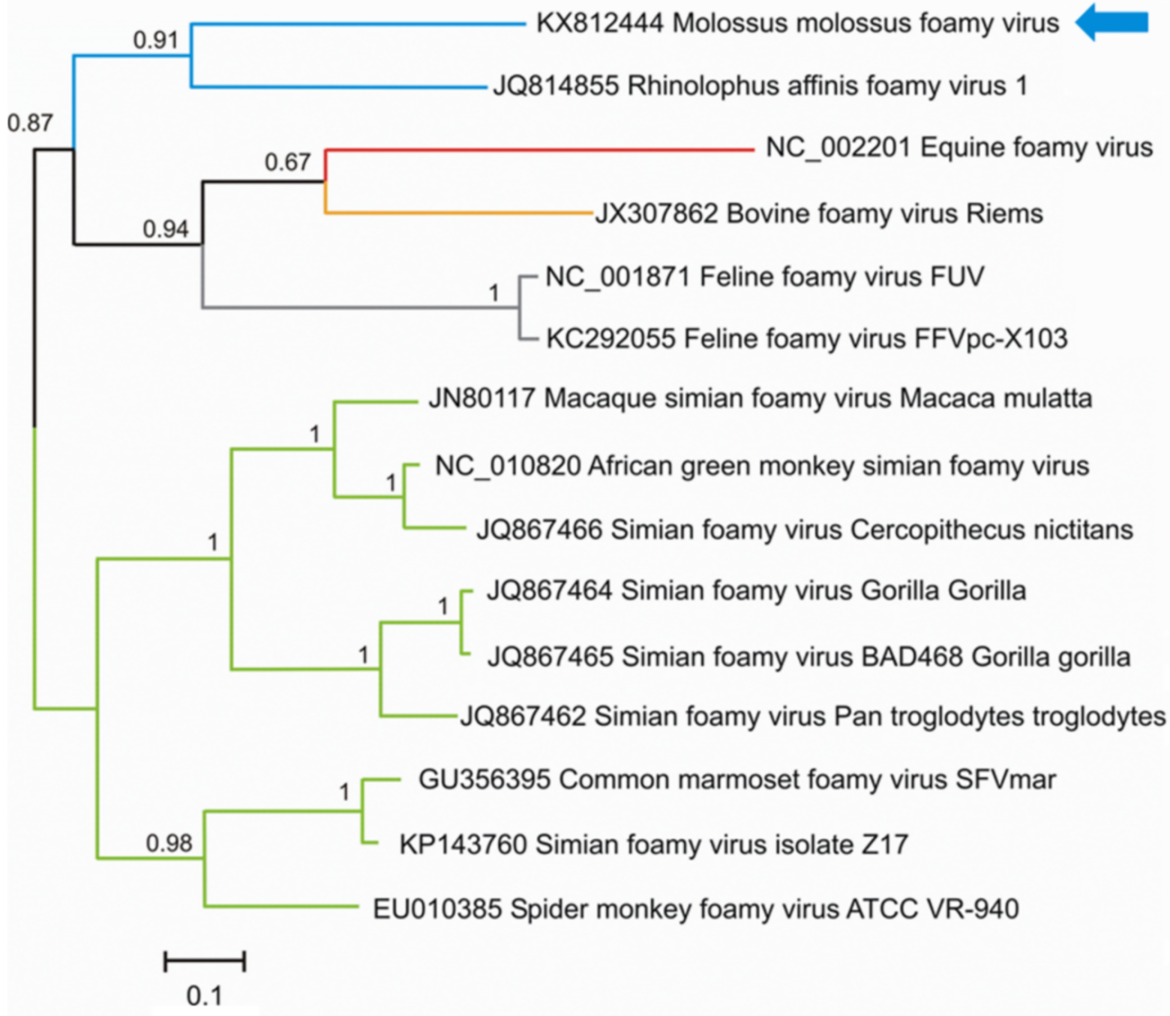
Phylogenetic analysis of partial sequences of the *pol* s region (alignment of 107 amino acids) directly obtained from the metagenomic data of pooled fecal samples of *M*. *molossus* with other representative members of the *Spumavirus* genus. The tree was inferred using the Bayesian method with the WAG + G model. Sequence identifiers include the NCBI accession number and the isolate name. The blue arrows indicate the sequence of bat-sourced spumavirus obtained in the present study. Posterior probabilities of the Bayesian analysis (>50%) are shown next to each node. The scale bar indicates amino acid substitutions per site.

*Spumavirus* (ICTV 2016) constitutes the only genus of the *Spumaretrovirinae* subfamily, which belongs to the *Retroviridae* family. They are highly prevalent in several animal species (*e*.*g*., cats, cows, horses and nonhuman primates) and currently six species have been described in the genus. Transmission of spumaviruses between nonhuman primates and cross-species transmission to humans occurs mainly through saliva (*e*.*g*., by licking, aggressive contacts, bites) [56–58]. Nevertheless, other routes of transmission, such as vertical transmission, are being studied [59–61]. In this study, a 312-nt-length fragment (Genbank acc. nb.: KX812444), obtained with two contigs detected in the saliva sample of *M*. *molossus* in urban habitats, showed 54.88% and 48.48% nucleotide and amino acid identity, respectively, with the *pol* gene of *Rhinolophus affinis foamy virus* 1 (Genbank acc. nb.: JQ814855) detected in the feces of Chinese bats [13]. Phylogenetic analysis revealed that the foamy virus (FV) identified was close to *Rhinolophus affinis foamy virus* 1, with a high posterior probability value of 0.91 (Fig 4). Bat FVs seem to share a common origin with equine, bovine and feline FVs. In addition, this analysis shows that bat FVs are divergent from those infecting nonhuman primates.

#### Bat herpesviruses

**Fig 5:**
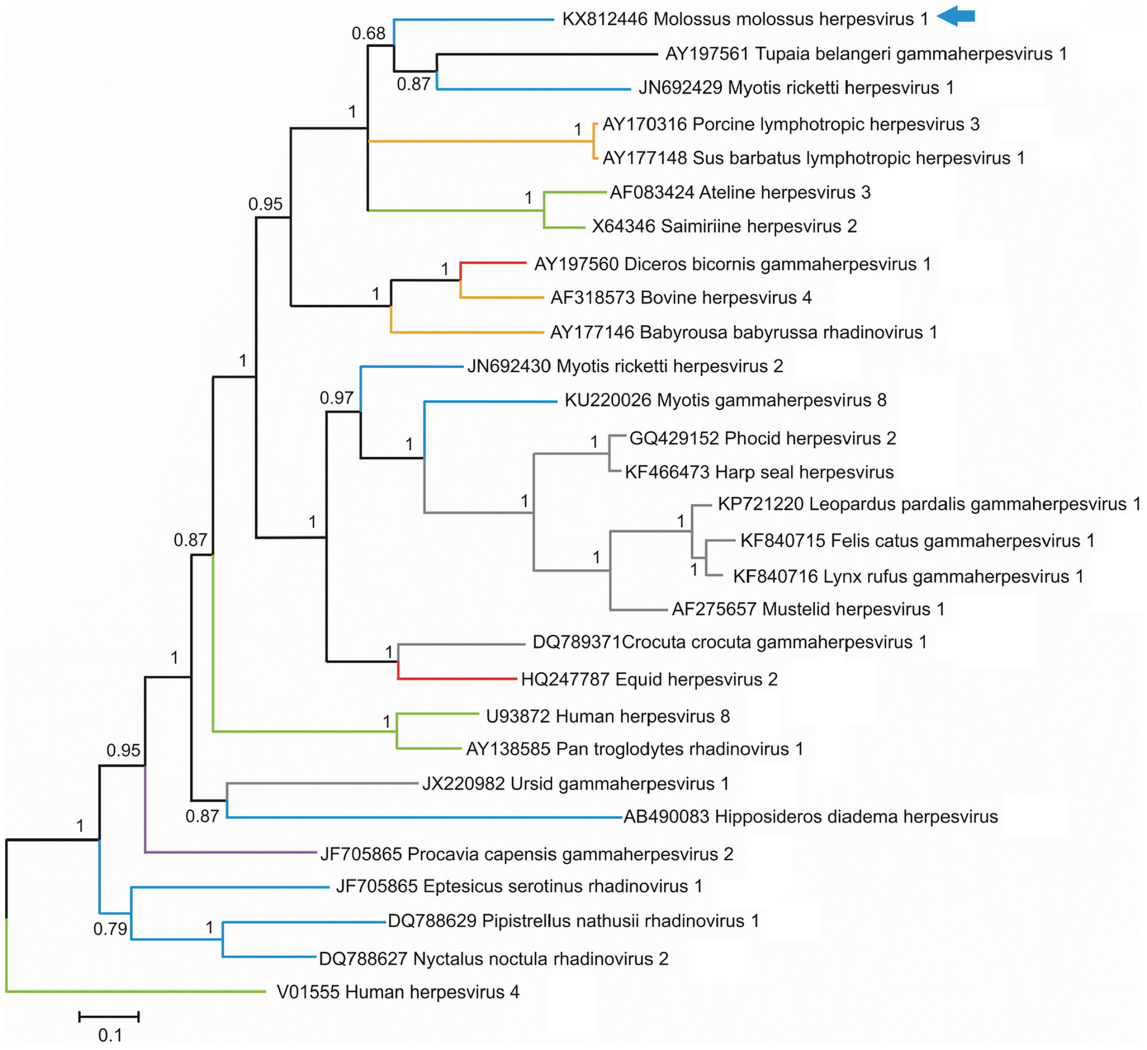
Phylogenetic analysis of partial *pol* gene sequences (alignment of 951 nucleotides) directly obtained from the metagenomic data of pooled fecal samples of *M*. *molossus* with other representative members of the *Herpesviridae* family. The tree was inferred using the Bayesian method with the GTR + G + I model. Sequence identifiers include the NCBI accession number and the isolate name. The blue arrows indicate the bat-sourced herpesvirus obtained in the present study. Posterior probabilities of the Bayesian analysis (>50%) are shown next to each node. The scale bar indicates amino acid substitutions per site.

Mammalian herpesviruses (HVs) belong to the *Herpesviridae* family (order *Herpesvirales*). HVs are organized in three subfamilies–*Alphaherpesvirinae*, *Betaherpesvirinae* and *Gammaherpesvirinae*. HVs can infect a wide range of hosts, including humans. Most HV-related contigs were found in the saliva sample of *M*. *molossus* in forest habitats. We identified a 943-nt-length fragment covering the *DNA polymerase* gene (Genbank acc. nb.: KX812446). This fragment displayed from 62.46% to 71.31% and from 68.93% to 80.58% nucleotide and amino acid identities, respectively, with other bat HVs. The highest percentage of identity in amino acids was observed with *Myotis ricketti herpesvirus 1* (Genbank acc. nb.: JN692429). Phylogenetic analysis showed that the HV identified belonged to the *Gammaherpesvirinae* subfamily and was related to the *Tupaia belangeri gammaherpesvirus* 1 and the *Myotis ricketti herpesvirus* 1, with a posterior probability of 0.68 (Fig 5).

#### Bat nairoviruses

**Fig 6:**
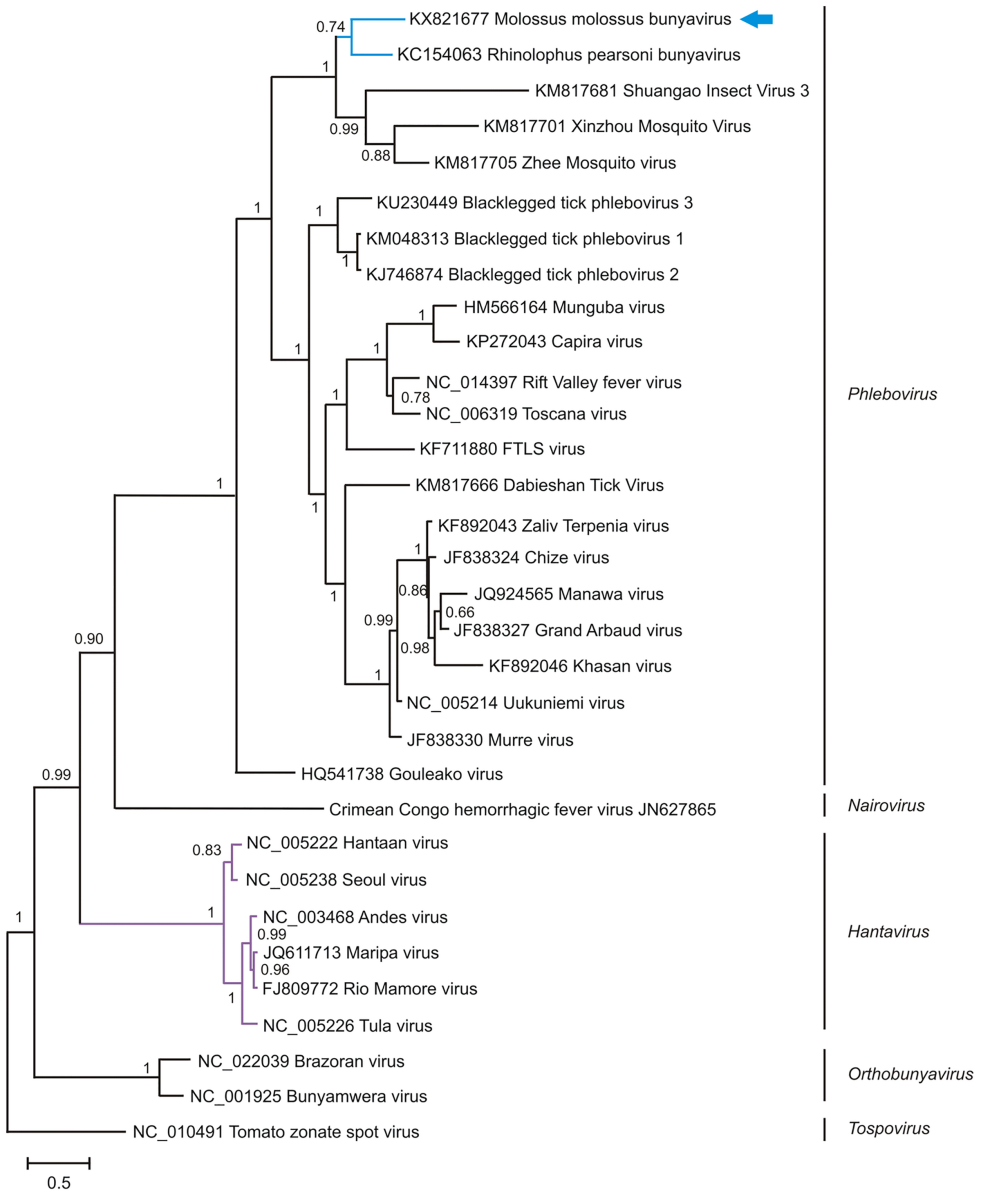
Phylogenetic analysis of partial RdRp protein sequences (alignment of 341 amino acids) directly obtained from the metagenomic data of pooled fecal samples of *M*. *molossus* with other representative members of the *Nairoviridae* family. The tree was inferred using the Bayesian method with the WAG + G model. Sequence identifiers include the NCBI accession number and the isolate name. The blue arrows indicate the bat-sourced nairovirus sequence obtained in the present study. Posterior probabilities of the Bayesian analysis (>50%) are shown next to each node. The scale bar indicates amino acid substitutions per site.

The *Nairoviridae* family belongs to the order *Bunyavirales* (ICTV 2016). This family encompasses at least nine serogroups, including the Crimean-Congo hemorrhagic fever serogroup. Nairoviruses (NVs) are primarily tick-borne viruses capable of infecting different vertebrate hosts. Four contigs matching nairoviruses (NVs) were detected in the feces of *M*. *molossus* in urban areas (Table 2). The contigs covered 1 kb of segment L (Genbank acc. nb: KX821677) and showed a 43% pairwise homology in amino acids with the RNA-dependent RNA polymerase of the previously described *Rhinolophus pearsoni bunyavirus* (Genbank acc. nb.: KC154063). Phylogenetic analysis revealed that the NV identified clustered with *Rhinolophus pearsoni bunyavirus*, with a posterior probability of 0.74 (Fig 6). These NVs share a common ancestor with mosquito NVs, with whom they clustered with a posterior probability of 1.

#### Bat papillomaviruses

**Fig 7:**
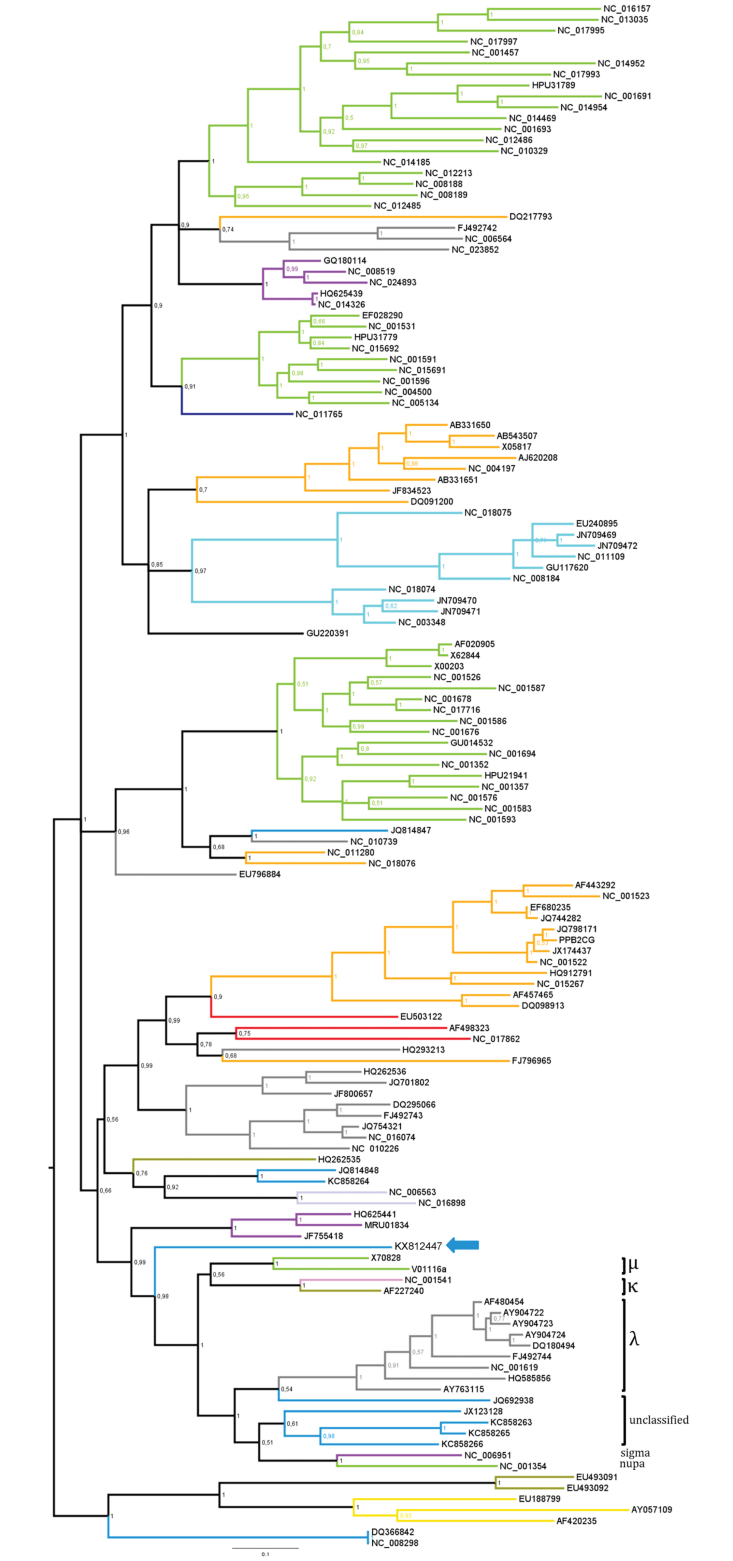
Phylogenetic analysis of the complete L1 protein sequence (alignment of 459 amino acids) directly obtained from the metagenomic data of pooled fecal samples of *M*. *molossus* with other representative members of the *Papillomaviridae* family. The tree was inferred using the Bayesian method with the WAG + G model. Sequence identifiers include the NCBI accession number. The blue arrows indicate the bat-sourced papillomavirus sequence obtained in the present study. Posterior probabilities of the Bayesian analysis (>50%) are shown next to each node. The scale bar indicates amino acid substitutions per site.

*Papillomaviridae* are nonenveloped dsDNA viruses. Currently, 49 genera have been identified in this family, including the most species-abundant genera *Gammapapillomavirus*. Papillomaviruses (PVs) are usually asymptomatic and thought to be rarely transmitted between species. In this study, a total of 235 contigs related to PVs were detected in *M*. *molossus* samples, with most of them identified in the feces collected in forest habitats (Table 2). The longest contig obtained covered the entire genome of known PVs and was named MmoPV1 (Genbank acc. nb.: KX812447). The MmoPV1 genome was 7,869 bp in length with a 40% G+C content. The MmoPV1 genome had the typical organization of PV ORFs (S2 Fig). The phylogenetic tree indicated that MmoPV1 possessed a basal position of a cluster of PVs encompassing mupa PVs, kappa PVs, lambda PVs, sigma PVs, nupa PVs and novel unclassified bat PVs detected in *Miniopterus schreibersii* [12], *Eidolon helvum* (Germany), *Eptesicus serotinus* and *Rhinolophus ferrumequinum* [62], with a high posterior probability value of 0.98 (Fig 7).

## Discussion

A wide spectrum of viruses capable of infecting a wide range of animals, plants, insects and bacteria was detected in bats. Since the 1950s, 28 viral families including more than 50 genera have been described in bats [18]. With the advent of new-generation technologies, the characterization of bat viromes and the discovery of related variants of known viruses and new bat-borne viruses continue to increase rapidly. The key purpose of the present work was to explore the viral composition of feces and saliva samples of *Desmodus rotundus* and *Molossus molossus* bat species, living in sympatry but using distinct microhabitats in French Guiana.

Overall, this study identified 10,991 viral-associated contigs distributed within 51 known viral families of which 14 are known to infect mammals. New mammalian-related viral sequences/variants were discovered, adding knowledge on the bat-borne viral population. The proportion of eukaryote- and prokaryote-related sequences was low (less than 2% and 20%, respectively). About 0.30% of total contigs were virus-related sequences, with differences in composition depending on the type of sample, the investigated species, habitats, and fine-scale ecological heterogeneity (Table E in S1 File). For instance, of the 30 viral families detected in *D*. *rotundus*, only 20 were common to both sampling sites (cave F *vs*. cave M). Likewise, focusing on vertebrate-related viral families, eight of the 11 viral families detected for this species were shared between the populations sampled in the two caves. For the *M*. *molossus* species, of the 46 viral families detected, 29 were common to both habitats (forest areas *vs*. urban areas). Focusing on vertebrate-related viral families, ten of the 12 viral families detected in this species were common to both habitats. Furthermore, we observed differences in the viral genera and species detected, depending on the sampling sites for both species. These results suggest that habitats may play a role in shaping the viral diversity harbored by the bats investigated. This observation was also supported by Hu *et al*. [16], who showed that the metagenomic approach can reflect differences in viral diversity carried by different bats in different regions. However, a verification of confounding factors (*e*.*g*., sampling size, type of sample, capture effort, species, feeding strategies) will be necessary to confirm these results. Likewise, population structure analysis at the species level and studies on interactions with other bat species using the same habitat are needed to determine their respective roles in shaping viral diversity.

Regardless of the bat species and the habitat studied, phage-related sequences represented a significant proportion of the viral-associated sequences identified (Table 2 and Table F in S1 File). Sequences related to the order *Caudovirales* and the *Microviridae* family were the most frequent. Phage-related sequences detected in the feces accounted for most of this proportion, as could be expected considering the type of sample. Despite the constant and rapid expansion of existing databases, sequences lacking counterparts remained the largest part of the data. These global results are in agreement with previous virome studies conducted on North American and Eurasian bat species [8,9,11,12,14,15] and highlight the need to pursue investigations and taxonomic assignments on microorganism communities. Therefore, the underestimation of the total number of bat-borne viruses present in the samples, including highly divergent viruses, cannot be excluded.

Despite a smaller number of samples for *M*. *molossus*, the number of viral-associated contigs detected in these samples was higher than that found in *D*. *rotundus*, suggesting a higher number of viruses passing through this species. However, these viruses are likely related to nondigested viruses associated with the diet of their prey but not directly to the host. Indeed, as highlighted in Li *et al*., insect and plant viruses were mostly represented in the insectivorous species, while they accounted for less than 5% in the vampire species [9]. The diet of the vampire bat (vertebrate blood) may also be reflected in the high proportion of vertebrate-related viral sequences found. Likewise, the divergence observed in the distribution of phage-related viruses might be associated with the diets of the species investigated and/or to their phylogenetic position, Molossidae *vs*. Phyllostomidae. For instance, *Podoviridae* species were the most frequent in *D*. *rotundus*, whatever the sampling site, while *Microviridae* species were the most often found in *M*. *molossus*. In bats, former studies highlighted a strong association between the microbiota, host phylogeny, life history, physiology as well as locality [63,64]. Given that gut microbiota is known to be highly dependent on diet, these results may reflect differences between the dietary ecology of *D*. *rotundus* and *M*. *molossus* species.

We found several novel mammalian-related viral sequences/variants from the *Anelloviridae*, *Nairoviridae*, *Circoviridae*, *Hepeviridae*, *Herpesviridae*, *Retroviridae* and *Papillomaviridae* families. These viral sequences were closely related to sequences detected in Old World bat species [17,62,65–68]. Furthermore, most of the families identified in Old World bat species were identified here, even for non-mammalian viruses. Moreover, where divergence could be expected due to geographical isolation, we observed highly supported phylogenetic proximity between Old and New World bat viruses in their respective continents, revealing the existence of common evolutionary processes and supporting the long evolutionary hypothesis of bats and their viruses [7]. For example, most of the circovirus (CV) sequences detected in the present study shared high sequence identities with bat CVs detected in several bat species trapped in different Chinese provinces [17,69] and clustered in highly supported monophyletic groups. The results reported herein suggest that the associated viruses may be hosted by bats. Conversely, the highly supported clustering of *M*. *molossus* CV (KX812443) with avian CVs may result from a cross-species jump from birds to bats, as reported by Hu *et al*. [16] and Lima *et al*. [70]. Likewise, foamy virus (FV) sequences, also called spumaviruses, detected in saliva swab samples of *M*. *molossus* showed a high genomic similarity with the FVs detected in pharyngeal and rectal swab samples of *Rhinolophus affinis* reported by Wu *et al*. [68], but low similarity with other spumaviruses infecting other hosts (*e*.*g*., equines, felines, bovines, nonhuman primates). Our findings indicate that FVs can indeed infect bats, which could be considered as potential reservoirs and dispersers of this viral genus.

Several studies reported the detection of herpesviruses (HVs) of the three subfamilies (*Alpha-*, *Beta-* and *Gammaherpesvirinae*) in feces, anal swabs, digestive tract and saliva samples of bats [16,17,68,71–77]. These findings suggested that the oral–fecal transmission route may be important for the transmission of HVs in bats. Furthermore, cases of cross-species transmission of HVs were also reported in bats, but their mode of transmission is still unclear [77]. In this study, we found a high diversity of HVs in the feces and saliva samples of both species investigated. Moreover, some sequences detected in the saliva samples of *M*. *molossus* from forest habitats were also found in the feces samples, which showed the capacity of these bats to shed HVs in the environment and supported the hypothesis of a potential oral–fecal transmission route. Detected HV-related sequences were novel and showed a high sequence similarity with other bat HVs. Nevertheless, the phylogenetic tree constructed using a Bayesian method revealed that *M*. *molossus herpesvirus 1* (KX812446) did not cluster in a unique group with other known bat gamma-HVs. Rather, it shares a common ancestor with *Myotis ricketti* and *Tupaia belangeri* gamma-HVs, stressing the need for further characterization of *M*. *molossus herpesvirus 1* to confirm its taxonomic assignment.

Nairoviruses (NVs) naturally infecting bats were reported in a few studies, with some capable of causing intestine and hepatic disorders in other mammals [15,78–80]. Evidence of neutralizing antibodies against Crimean-Congo hemorrhagic fever (CCHF) was reported by Müller *et al*. [81], but the role of bats regarding the cycle and dispersal of this virus is not clear. Here, we found a novel NV-related sequence in the feces of *M*. *molossus* sampled in urban areas. Phylogenetic relationships based on the partial segment L sequence showed a highly supported clustering with another NV-related sequence detected from the feces of *Rhinolophus pearsoni*. This analysis also revealed that the detected NV was related to the genus *Phlebovirus*, primarily constituted of tick-, mosquito- and phlebotomine-borne viruses. These findings suggest that this sequence may be related to undigested viruses associated with the bats’ diet rather than viruses hosted by bats. However, screening of other organs will be necessary to rule out the role of bats in the virus cycle and dispersal.

Diverse sequences related to papillomaviruses (PVs) were reported in bats [16,17,62,66,67,82,83]. Here, we detected 235 PV-like sequences related to members of genetically diverse genera within the *Papillomaviridae*, indicating that bat species might be associated with a wide diversity of PVs. We described the complete genome of a novel bat PV, MmoPV1 (KX812447), detected in the feces and saliva samples of *M*. *molossus* sampled in forest and urban habitats. This is the first detection of PV in this species to date. Comparing MmoPV1 with other PVs showed that it contains the typical PV ORFs coding for four putative early proteins (E6, E7, E1, E2) and two putative late capsid proteins (L2 and L1). The putative early protein E4 –present in other phylogenetically closely-related bat PVs (JQ692938 [67], JX123128 [83], KC858263, KC858265, KC858266 [84])–was absent in MmoPV1. Furthermore, the phylogenetic tree revealed that MmoPV1 may be more ancient than these bat PVs, suggesting that they may either have undergone a divergent evolution that allowed them to acquire the early protein or represent different host-specific and area-specific lineages [84].

This study is the very first to explore the virome of fecal and saliva samples obtained from the two common Amazonian bat species, *M*. *molossus* and *D*. *rotundus*, with overlapping distribution but using distinct microhabitats in French Guiana. It provides important insight into the feces and saliva viromes of the two bat species investigated within contrasting habitats.

However, to fully understand how viral diversity is shaped in *D*. *rotundus* and *M*. *molossus*, further characterization of their geographic range, foraging-induced interactions, species distribution and gregariousness will be necessary. Indeed, larger geographic ranges encompass greater diversity of ecosystems/habitats, which could lead to a spatial population substructure [35]. The overlapping distribution of reservoirs and recipient hosts, the social complexity of bats as well as species-specific evolutionary and life-history traits are known as predictors of viral richness in wildlife [6,35]. Likewise, the contact and proximity between individuals influence the presence, abundance and diversity of the pathogens harbored [85]. Consequently, deciphering the virome of bats should consider the size of the whole community of bats, the genetic structure of populations in a given habitat and the rates of interactions among them, all parameters contributing to the viral diversity observed for bat species.

## Supporting information

S1 File Read and contig processing of *D*. *rotundus* and *M*. *molossus* samples. Read data reduction steps and d*e novo* assembly of processed reads. Contig and sequential BLAST comparisons, with the total number of contigs and viral families identified in *D*. *rotundus* and *M*. *molossus* samples.
(DOCX)

S1 Fig Map of collecting sites of all samples/individuals used across French Guiana. The total number of collected samples per site is given in parentheses. Pie chart indicates the proportion of feces (orange) and saliva (orange dots) samples collected. Detailed characteristics of the different collecting sites are given in Table 1.
(TIF)

S2 Fig Predicted genome organization of the putative papillomavirus sequence directly recovered from the metagenomic data of *M*. *molossus* bat feces. The open-reading frames encoding the putative late (L1 and L2) and early (E1, E2, E6 and E7) proteins are shown in green boxes. The genome organization was determined with Geneious R9.
(TIF)
