# Supplementary material for: Virome analysis of two sympatric bat species (Desmodus rotundus and Molossus molossus) in French Guiana
Source: PLoS One. 2017 Nov 8;12(11):e0186943. doi: 10.1371/journal.pone.0186943 (PMC5695591; doi:10.1371/journal.pone.0186943)
Supplement: S1 File — Read data reduction steps and de novo assembly of processed reads. Contig and sequential BLAST comparisons, with the total number of contigs and viral families identified in D. rotundus and M. molossus samples. (DOCX) [file pone.0186943.s001.docx]

**Table A. Read data processing and reduction steps.**

|  |  |  |  |  |  | **Data reduction steps** | |
| --- | --- | --- | --- | --- | --- | --- | --- |
| **Species** | **Habitats** | **Sites** | **Samples** | ***n*** | ***raw*** | ***ndn*** | ***dn*** |
| *D. rotundus* | Forest | Cave F | Feces | 75 | 7 752 462 | 2 536 419 | 903 760 |
|  |  | Cave M | Saliva | 50 | 8 820 103 | 948 448 | 283 641 |
|  |  |  | Feces | 66 | 3 419 472 | 2 227 250 | 1 174 147 |
| *M. molossus* | Forest | Paracou Saut Athanase | Saliva | 58 | 9 642 567 | 1 741 389 | 718 421 |
|  |  |  | Feces | 14 | 7 683 786 | 1 318 143 | 606 678 |
|  | Urban | Cacao La Chaumière | Saliva | 30 | 8 206 846 | 555 174 | 145 005 |
|  |  |  | Feces | 5 | 7 800 358 | 1 012 929 | 285 892 |
|  |  |  | **Total** | **298** | **53 325 594** | **10 339 752** | **4 117 544** |

The number of individuals (***n***), the number of raw reads obtained (***raw***), the total number of reads after trimming and cleaning (***ndn***) and the number of reads after digital normalization (***dn***) are given per sample.

**Table B. *De novo* assembly of processed read data comparison 1 between k-mers.**

| **Species** | **Habitats** | **Sites** | **Samples** | **Assembler** | ***ndn*** | ***ndn* ctds 1** | ***ndn* % saved** | ***dn*** | ***dn* ctds 1** | ***dn* % saved** |
| --- | --- | --- | --- | --- | --- | --- | --- | --- | --- | --- |
| *D. rotundus* | Forest | Cave F | Feces | SPAdes | 906 221 | 502 668 | 55.47 | 857 888 | 487 271 | 56.80 |
|  |  |  |  | Velvet | 830 383 | 455 449 | 54.85 | 760 331 | 447 250 | 58.82 |
|  |  | Cave M | Feces | SPAdes | 1 563 094 | 893 042 | 57.13 | 1 487 086 | 864 105 | 58.11 |
|  |  |  |  | Velvet | 1 780 403 | 980 990 | 55.10 | 1 674 112 | 902 296 | 53.90 |
|  |  |  | Saliva | SPAdes | 177 600 | 89 194 | 50.22 | 159 413 | 82 626 | 51.83 |
|  |  |  |  | Velvet | 154 256 | 78 787 | 51.08 | 139 110 | 77 069 | 55.40 |
| *M. molossus* | Forest | Paracou Saut Athanase | Feces | SPAdes | 622 208 | 364 291 | 58.55 | 594 271 | 354 777 | 59.70 |
|  |  |  |  | Velvet | 866 316 | 483 006 | 55.75 | 826 353 | 475 251 | 57.51 |
|  |  |  | Saliva | SPAdes | 680 049 | 402 036 | 59.12 | 652 835 | 392 165 | 60.07 |
|  |  |  |  | Velvet | 706 568 | 389 182 | 55.08 | 491 700 | 313 242 | 63.71 |
|  | Urban | Cacao La Chaumière | Feces | SPAdes | 138 947 | 76 737 | 55.23 | 124 906 | 71 236 | 57.03 |
|  |  |  |  | Velvet | 106 053 | 67 958 | 64.08 | 92 011 | 62 257 | 67.66 |
|  |  |  | Saliva | SPAdes | 80 184 | 44 642 | 55.67 | 72 344 | 40 609 | 56.13 |
|  |  |  |  | Velvet | 68 872 | 35 497 | 51.54 | 61 178 | 34 524 | 56.43 |

Contigs generated using the k-mers 21, 55 and 99 were pooled (contigs generated from ***ndn***, non-digitally normalized reads and ***dn***, digitally normalized reads). The total number of contigs retained after removal of duplicates and clustering with a 95% threshold identity value (***ndn*** **ctds 1** and ***dn*** **ctds 1**) as well as the percentage of data saved are given for each data set and each assembler (SPAdes and Velvet).

**Table C. *De novo* assembly of processed read data, comparison 2 between SPAdes and Velvet assemblers.**

| **Species** | **Habitats** | **Sites** | **Samples** | **Assembler** | ***ndn* ctds 1** | ***ndn* ctds 2** | ***ndn* % saved** | ***dn* ctds 1** | ***dn* ctds 2** | ***dn* % saved** |
| --- | --- | --- | --- | --- | --- | --- | --- | --- | --- | --- |
| *D. rotundus* | Forest | Cave F | Feces | SPAdes + Velvet | 958 117 | 669 997 | 69.93 | 934 521 | 653 908 | 69.97 |
|  |  | Cave M | Feces | SPAdes + Velvet | 1 874 032 | 1 323 490 | 70.62 | 1 766 401 | 1 263 098 | 71.51 |
|  |  |  | Saliva | SPAdes + Velvet | 167 981 | 117 914 | 70.19 | 159 695 | 113 587 | 71.13 |
| *M. molossus* | Forest | Paracou Saut Athanase | Feces | SPAdes + Velvet | 847 297 | 594 618 | 70.18 | 830 028 | 581 658 | 70.08 |
|  |  |  | Saliva | SPAdes + Velvet | 791 218 | 538 768 | 68.09 | 705 407 | 503 908 | 71.44 |
|  | Urban | Cacao La Chaumière | Feces | SPAdes + Velvet | 144 695 | 105 896 | 73.19 | 133 493 | 97 325 | 72.91 |
|  |  |  | Saliva | SPAdes + Velvet | 80 139 | 55 373 | 69.10 | 75 133 | 52 067 | 69.30 |

Contigs from comparison 1 were pooled (***ndn* ctds 1** and ***dn* ctds 1**). The number of contigs retained after removal of duplicates and clustering with 95% a threshold identity value (***ndn*** **ctds** **2** and ***dn*** **ctds 2**) as well as the percentage of data saved are given for each data set.

**Table D. *De novo* assembly of processed read data, comparison 3 between contigs generated with digitally normalized and non-digitally normalized reads.**

| **Species** | **Habitats** | **Sites** | **Samples** | ***ndn* + *dn* (ctds 2)** | **Centroids** | ***ndn* + *dn* % saved** |
| --- | --- | --- | --- | --- | --- | --- |
| *D. rotundus* | Forest | Cave F | Feces | 1 323 905 | 669 997 | 50.61 |
|  |  | Cave M | Feces | 2 586 588 | 1 323 490 | 51.17 |
|  |  |  | Saliva | 231 501 | 117 914 | 50.93 |
| *M. molossus* | Forest | Paracou Saut Athanase | Feces | 1 176 276 | 648 927 | 55.17 |
|  |  |  | Saliva | 1 042 676 | 569 059 | 54.58 |
|  | Urban | Cacao La Chaumière | Feces | 203 221 | 114 421 | 56.30 |
|  |  |  | Saliva | 107 440 | 59 544 | 55.42 |

Contigs from comparison 2 were pooled (***ndn*** + ***dn*** **(ctds 2)**). The number of contigs retained after removal of duplicates and clustering with a 95% threshold identity value (**centroids**) as well as the percentage of data saved are given for each data set.

**Table E. Sequential BLAST comparisons.**

|  |  |  |  |  |  | **BLAST match** | | | | | | | |
| --- | --- | --- | --- | --- | --- | --- | --- | --- | --- | --- | --- | --- | --- |
| **Species** | **Habitats** | **Sites** | **Samples** | ***n*** | **Centroids** | ***Eukaryota*** | | ***Bacteria*** | | ***Virus*** | | **No hit** | |
| *D. rotundus* | Forest | Cave F | Feces | 75 | 725 786 | 5 017 | (0.69%) | 235 | (4.68%) | 1 929 | (0.27%) | 718 605 | (99.01%) |
|  |  | Cave M | Saliva | 50 | 134 319 | 1 237 | (0.92%) | 243 | (19.64%) | 40 | (0.03%) | 132 799 | (98.87%) |
|  |  |  | Feces | 66 | 1 470 163 | 25 574 | (1.74%) | 192 | (0.75%) | 2 551 | (0.17%) | 1 441 846 | (98.07%) |
| *M. molossus* | Forest | Paracou Saut Athanase | Saliva | 58 | 569 059 | 2 126 | (0.37%) | 114 | (5.36%) | 729 | (0.13%) | 566 090 | (99.48%) |
|  |  |  | Feces | 14 | 648 927 | 2 660 | (0.41%) | 488 | (18.35%) | 3 039 | (0.47%) | 642 740 | (99.05%) |
|  | Urban | Cacao La Chaumière | Saliva | 30 | 59 544 | 1 010 | (1.70%) | 113 | (11.19%) | 8 | (0.01%) | 58 413 | (98.10%) |
|  |  |  | Feces | 5 | 114 421 | 464 | (0.41%) | 70 | (15.09%) | 2 687 | (2.35%) | 111 200 | (97.18%) |
|  |  |  | **Total** | **298** | **3 722 219** | **38 088** | **(1.02%)** | **1 455** | **(3.82%)** | **10 983** | **(0.30%)** | **3 671 692** | **(98.64%)** |

The number of samples collected (***n***), contigs retained from comparison 3 (**centroids**), the number of contigs matching ***Eukaryota***, ***Bacteria*** and ***Virus***, as well as the total number of negative contigs (**no hit**) on both nucleotide and protein homology searches, are given for each data set. The representative percentage of each taxonomic group from the centroids is given in parenthesis for each data set.

**Table F. Total number of contigs and viral families identified in *D. rotundus* and *M. molossus*.**

|  |  | **Saliva** | | | |  | **Feces** | | | |  | **Overall** | | | |
| --- | --- | --- | --- | --- | --- | --- | --- | --- | --- | --- | --- | --- | --- | --- | --- |
|  | **Natural host** | **Cave F** | **Cave M** | **Urban** | **Forest** |  | **Cave F** | **Cave M** | **Urban** | **Forest** |  | **Cave F** | **Cave M** | **Urban** | **Forest** |
| **A.** | Insect virus |  | 2 | 0 | 27 |  | 3 | 7 | 664 | 296 |  | 3 | 9 | 664 | 323 |
|  | Phage |  | 3 | 2 | 52 |  | 1 209 | 1 779 | 1 427 | 1 927 |  | 1 209 | 1 782 | 1 429 | 1 979 |
|  | Plant and protozoan virus |  | 3 | 0 | 0 |  | 19 | 15 | 90 | 80 |  | 19 | 18 | 90 | 80 |
|  | Vertebrate virus |  | 31 | 4 | 646 |  | 599 | 677 | 264 | 390 |  | 599 | 708 | 268 | 1 036 |
|  | Unknown |  | 0 | 2 | 4 |  | 100 | 76 | 247 | 346 |  | 100 | 76 | 249 | 350 |
|  | **Total** |  | **39** | **8** | **729** |  | **1 930** | **2 554** | **2 692** | **3 039** |  | **1 930** | **2 593** | **2 700** | **3 768** |
|  |  |  |  |  |  |  |  |  |  |  |  |  |  |  |  |
| **B.** | Insect virus |  | 1 | 0 | 3 |  | 2 | 4 | 9 | 10 |  | 2 | 4 | 9 | 12 |
|  | Phage |  | 2 | 1 | 4 |  | 8 | 6 | 6 | 5 |  | 8 | 6 | 6 | 5 |
|  | Plant and protozoan virus |  | 2 | 0 | 0 |  | 4 | 5 | 11 | 10 |  | 4 | 7 | 11 | 10 |
|  | Vertebrate virus |  | 6 | 3 | 8 |  | 10 | 9 | 11 | 10 |  | 10 | 9 | 12 | 11 |
|  | Unknown |  |  |  |  |  |  |  |  |  |  |  |  |  |  |
|  | **Total** |  | **11** | **4** | **15** |  | **24** | **24** | **36*** | **34*** |  | **24** | **26** | **37*** | **37*** |

The total number of contigs (**A**) and viral families (**B**) identified in the study are given per sample (**saliva** and **feces**) and **overall**, for each species and habitats: caves F and M for *D. rotundus*, urban and forest for *M. molossus*. * The nature of the host which provided the samples from which the associated sequence was derived from is excluded: *Poxviridae* and *Parvoviridae* are counted once.
