# Supplementary figures and images for: Virome analysis of two sympatric bat species (Desmodus rotundus and Molossus molossus) in French Guiana
Source: PLoS One. 2017 Nov 8;12(11):e0186943. doi: 10.1371/journal.pone.0186943 (PMC5695591; doi:10.1371/journal.pone.0186943)

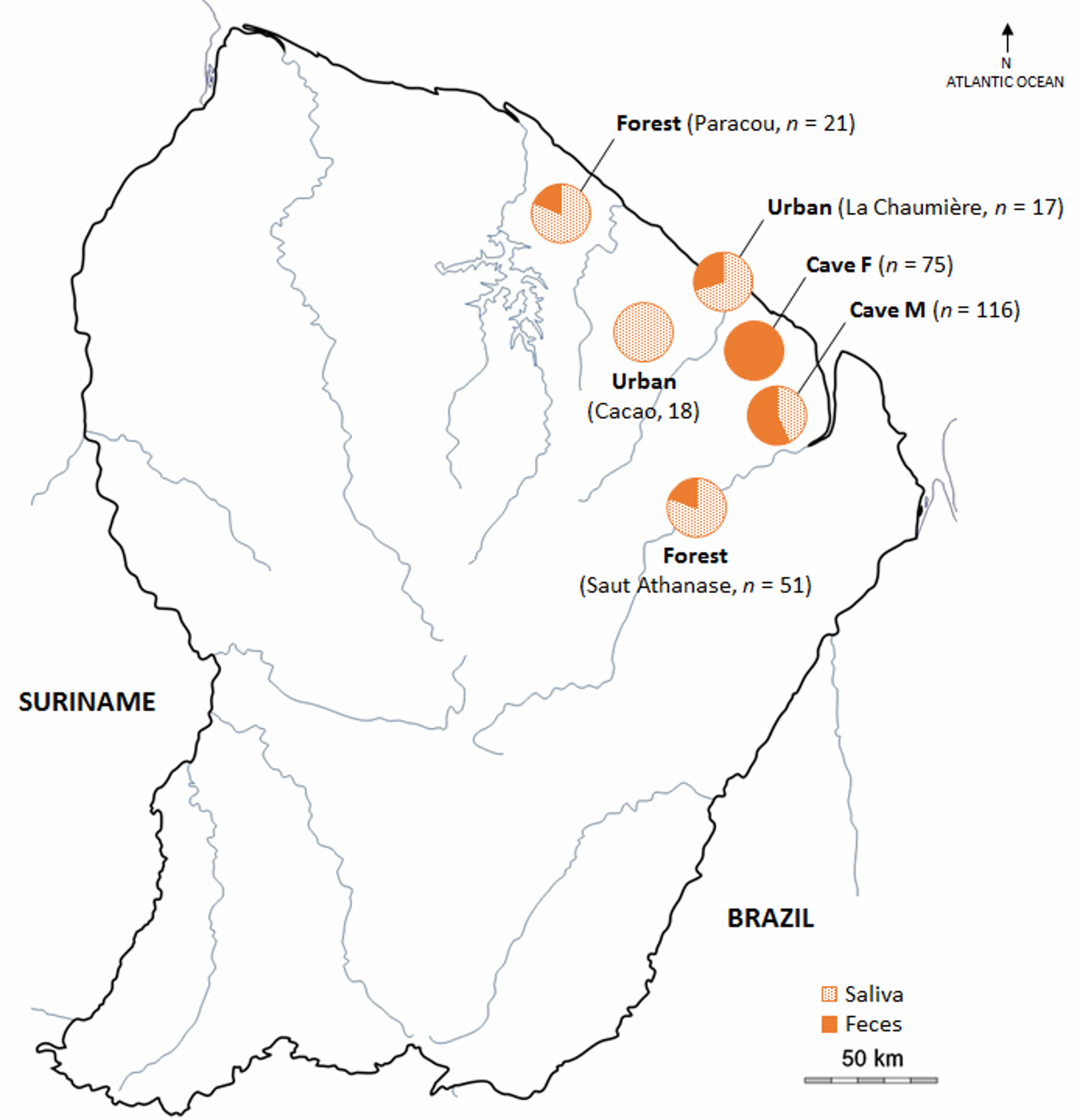

Supplement: S1 Fig — The total number of collected samples per site is given in parentheses. Pie chart indicates the proportion of feces (orange) and saliva (orange dots) samples collected. Detailed characteristics of the different collecting sites are given in Table 1. (TIF) [file pone.0186943.s002.tif]

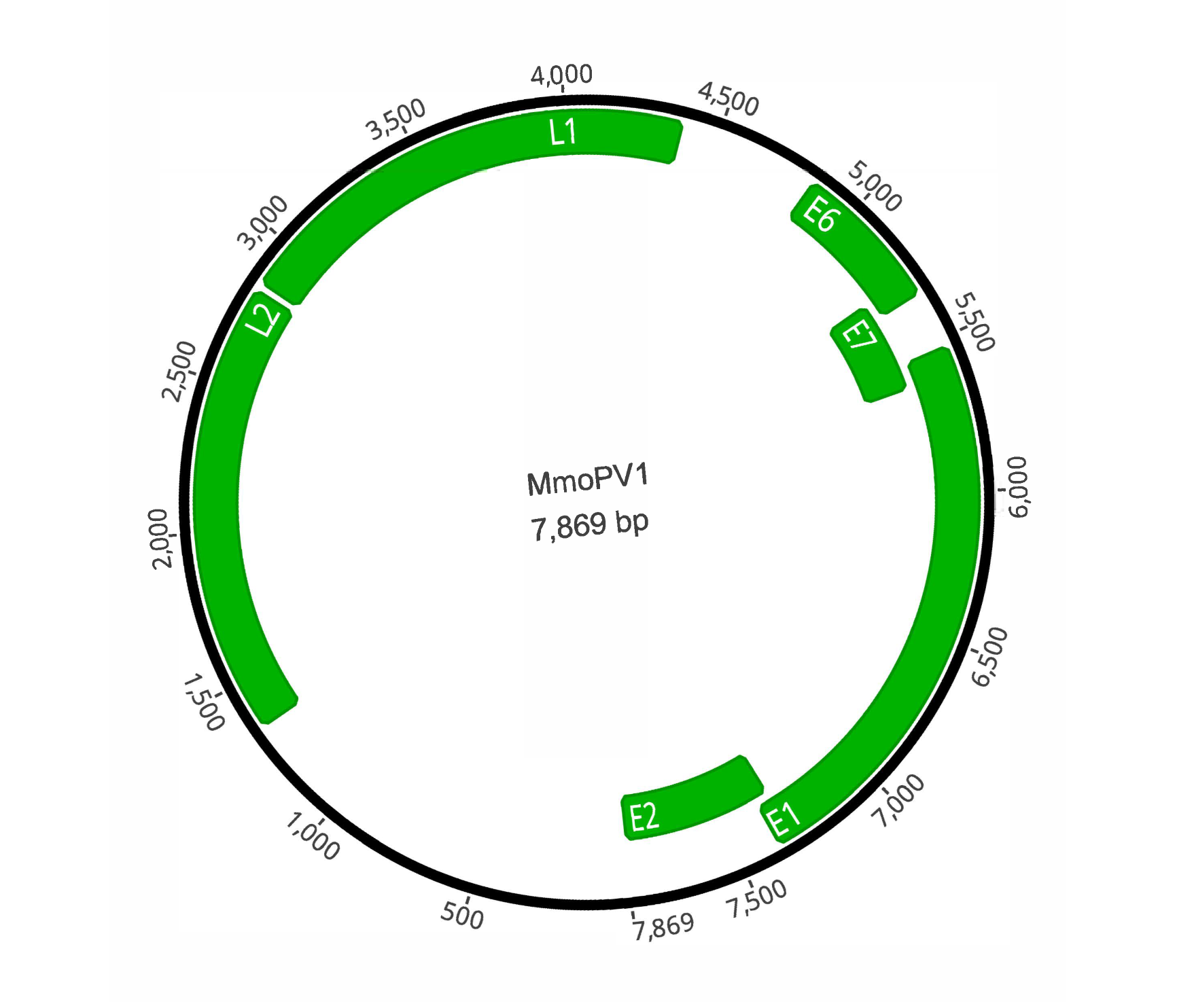

Supplement: S2 Fig — The open-reading frames encoding the putative late (L1 and L2) and early (E1, E2, E6 and E7) proteins are shown in green boxes. The genome organization was determined with Geneious R9. (TIF) [file pone.0186943.s003.tif]
